# Unlocking the Functional Value of European-Originated *Chrysanthemum* Hybrids: Phytochemical and Bioactivity Assessment

**DOI:** 10.3390/molecules31010172

**Published:** 2026-01-01

**Authors:** Natalia Miler, Maciej Balcerek, Jakub Gębalski, Anita Woźny, Magdalena Wójciak, Ireneusz Sowa, Daniel Załuski

**Affiliations:** 1 Laboratory of Horticulture and Landscape Architecture, Department of Biotechnology, Faculty of Agriculture and Biotechnology, Bydgoszcz University of Science and Technology, 85-796 Bydgoszcz, Poland; wozny@pbs.edu.pl; 2 Department of Pharmaceutical Botany and Pharmacognosy, Ludwik Rydygier Collegium Medicum, Nicolaus Copernicus University, 85-094 Bydgoszcz, Poland; balcerek@cm.umk.pl (M.B.); jakub.gebalski@cm.umk.pl (J.G.); daniel.zaluski@cm.umk.pl (D.Z.); 3 Department of Analytical Chemistry, Medical University of Lublin, 20-093 Lublin, Poland; magdalena.wojciak@umlub.edu.pl (M.W.); ireneusz.sowa@umlub.edu.pl (I.S.)

**Keywords:** herbal application, dry inflorescences, antioxidant activity, terpenoids, phenolic acids, flavonoids, pigments, enzyme inhibition

## Abstract

Chrysanthemums are appreciated not only for their ornamental and medicinal attributes but also as edible plants long incorporated into teas, infusions, and culinary traditions. Yet, hybrid cultivars specifically adapted to European growing conditions remain poorly characterized with respect to their medicinal potential. In this study, we investigated the phytochemical composition, antioxidant properties, and enzyme-inhibitory activities of inflorescences of four field-grown *Chrysanthemum* × *morifolium* ‘Donna’ × *C. rubellum* ‘Clara Curtis’ hybrids of European origin (CD 7, DC 19, DC 26, CD 46). Their profiles were compared with those of a Chinese tea cultivar (*C. morifolium* CHR18) and a commercial herbal product (CH B). Chemical constituents were analyzed using GC–MS and LC–MS, while antioxidant activity was evaluated by FRAP, CUPRAC, DPPH, ABTS, and iron-chelating assays; hyaluronidase (HYAL) and butyrylcholinesterase (BChE) inhibition were also assessed. A total of 61 volatile compounds were identified, with several terpenoids—such as chrysanthenone and verbenone—occurring exclusively in the European hybrids. CHR 18 possessed the highest flavonoid and phenolic acid levels, whereas hybrid CD 46 exhibited the most pronounced overall antioxidant performance. Hyaluronidase inhibition was strongest in DC 26 and CD 46 (60–62%), surpassing both reference samples, while BChE inhibition remained generally low. Overall, the results highlight that *C. morifolium* × *C. rubellum* hybrids developed for cultivation in the temperate European climate offer a unique combination of phytochemical richness, robust antioxidant activity, and noteworthy enzyme inhibition. These traits underscore their promise as emerging functional chrysanthemum resources and support future applications in European herbal products, nutraceutical development, and region-specific functional food innovation.

## 1. Introduction

Chrysanthemums (*Chrysanthemum* × *morifolium* Ramat.) are globally recognized ornamental plants, cultivated both for cut flowers and as potted plants. They consistently rank among the top-selling ornamentals worldwide. Although chrysanthemums are short-day plants, cultivation technologies make it possible to supply market-ready products year-round. Thanks to their remarkably rich and adaptable genome, as well as their susceptibility to hybridization, thousands of cultivars are grown around the world, differing in color, flower shape, growth habit, and vigor [1].

While in Europe and the Americas chrysanthemums are valued mainly for their ornamental qualities, their earliest documented use—dating back to the time of Confucius—was medicinal. In China, chrysanthemums remain an important component of Traditional Chinese Medicine (TCM) [2], and dried inflorescences are widely consumed as a health-promoting herbal infusion (Chinese: *Juhua*; Korean: *Gukhwa*; Japanese: *Kikku*) [3]. The Chinese Pharmacopoeia recognizes five traditional medicinal cultivars—‘Hangju’, ‘Boju’, ‘Gongju’, ‘Chuju’, and ‘Huaiju’—which vary in morphology, pigmentation, and regional origin [4]. Chrysanthemum inflorescences are used in TCM to alleviate symptoms such as headache, fever, red or irritated eyes, and inflammation [5], and modern pharmacological studies confirm their antimicrobial, anti-inflammatory, antioxidant, antiviral, antiadipogenic, and hepatoprotective activities [3,6,7,8].

In Western countries, however, there is no established tradition of growing chrysanthemums for herbal use. Historical limitations in trade with East Asia and the low ornamental value of traditional medicinal cultivars contributed to their limited introduction [9]. Contemporary European and North American cultivars are bred primarily for decorative purposes, and intensive production systems relying on pesticides and growth regulators further limit their suitability as herbal raw material [4]. This creates a notable gap: there is a growing interest in chrysanthemum-based herbal products outside Asia, but locally adapted cultivars suitable for clean-field, residue-free production are lacking.

The origin of *Chrysanthemum rubellum* remains somewhat unclear. It was recognized as a distinct species in 1938 in UK by a botanist John Sealy from Kew Gardens and used to create new perennial chrysanthemum hybrids that are still cultivated, like ‘Clara Curtis’ obtained by renowned breeder Amos Perry in the 1930s [10]. Today, it is believed that the Rubellum Group is more likely a progeny of a mutant form of *C. zawadskii* var. *latillobum*, although further genetic studies are needed to confirm this hypothesis [9]. Hybrids of *C. rubellum*, likely due to their cold-hardy ancestral lineage, possess a remarkable ability to survive even very low temperatures. In addition, they are scented—a sweet fragrance attracts bees and butterflies, a trait not observed in *C. morifolium*. Other noteworthy characteristics, such as early flowering and a wide palette of pastel shades in both single and double inflorescence forms, make these cultivars particularly appealing for landscape use [10]. Recent studies have demonstrated the antidiabetic potential of *C. rubellum* extracts in in vitro experiments [11] and their stimulatory effect on the proliferation of dental pulp stem cells [12]. These traits—cold-hardiness, early blooming, and emerging evidence of bioactivity—position *C. rubellum* and its hybrids as promising candidates for the development of herbal chrysanthemum cultivars adapted to the climatic conditions of Europe and other temperate regions.

The aim of this study was to investigate the phytochemical composition and activity of field-grown chrysanthemum hybrids, and to compare them with a reference cultivar of Chinese tea chrysanthemum as well as with commercially available crude herbal chrysanthemum material. The novelty of this study lies in evaluating whether hybrids between an ornamental cultivar (*Chrysanthemum × morifolium* ‘Donna’) and a cold-hardy, bioactive taxon (*C. rubellum* ‘Clara Curtis’) can serve as a new source of phytochemically rich and biologically active chrysanthemum material suitable for herbal applications. Our findings may contribute to the development of new functional beverages and dietary ingredients derived from chrysanthemum hybrids adapted to European growing conditions.

## 2. Results and Discussion

Plants synthesize secondary metabolites for functions beyond core metabolism. They act mainly as chemical defenses, stress protectants and internal/external signaling molecules supporting survival in variable environments [13]. Moreover, these compounds play an important role in human nutrition and health. Many plant secondary metabolites, such as polyphenols, alkaloids, terpenoids, and saponins, exhibit antioxidant, anti-inflammatory, antimicrobial, and anticancer properties. Through the regular dietary intake, they contribute to the prevention of chronic diseases, modulation of the immune system, and maintenance of metabolic balance. Consequently, biological activity of these metabolites is crucial not only for plant physiology but also for their potential application in functional foods and nutraceuticals [14].

Plants of the *Asteraceae* family are an exceptionally rich source of valuable secondary metabolites with applications in medicine, cosmetology, the food industry, and beyond [15]. Chrysanthemums, as members of this family, exhibit important properties arising from their biochemical composition, which have been valued for centuries by practitioners of traditional Chinese medicine and consumers across Asia [4,16]. To date, however, hybrids of *C.* × *morifolium* with *C. rubellum* have not been investigated for their phytochemical properties. Research of this kind—focusing on genotypes adapted to field cultivation under Europe’s temperate climate conditions and possessing valuable phytochemical properties—remains pioneering.

### 2.1. Terpenoid Diversity and Genotype-Specific Volatile Profiles in Chrysanthemum Flowers

Terpenoids, a substantial group of volatile organic compounds (VOCs) produced by various plant organs, play a particularly important role in plant defense systems against pests, and in the attraction of pollinators [17]. As volatiles, they also contribute to plant thermoregulation [18]. As aromatic compounds, terpenoids find broad application in the cosmetic and food industries. More interestingly, depending on their specific chemical structures, they may exhibit antimicrobial, anti-inflammatory, and/or antioxidant properties—although their antioxidant capacity is typically weaker than that of flavonoids [19]. In chrysanthemums, terpenoids are responsible for individual flavor of beverages based on *chrysanthemi flos* [8,20,21].

The Asteraceae family, to which the *Chrysanthemum* genus belong, is known for its abundance of species that are prolific producers of VOCs. In our experiments, in the studied group of chrysanthemum genotypes, a total of 61 volatile compounds were identified using GC-MS, including both terpenoids and several non-terpenoid volatile compounds commonly found in plants (Table 1). Among these, there were 10 monoterpenes, 25 monoterpenoids, 5 sesquiterpenes, 7 sesquiterpenoids, and 4 triterpenes. Noteworthy, only three compounds were detected in all genotypes: α-pinene, camphene, and alcanfor (also known as camphor).

There can be found species- and cultivar-specific differences in qualitative and quantitative results for terpenoids content in inflorescences of chrysanthemums. Genotype-specific production and emission of volatiles by different *Chrysanthemum morifolium* cultivars and wild relatives—including *C. lavandulifolium*, *C. nankingense*, *C. dichrum*, *C. zawadskii* var. *latilobum*, *C. vestitum*, and *C. indicum*—has been confirmed through detailed chemical analyses [8]. In a comprehensive study, a total of 46 terpenoid compounds were identified using GC-MS in flower head extracts. It was found that β-myrcene, β-elemene, β-cadinene, and β-caryophyllene were synthesized specifically by wild relatives such as *C. vestitum* and *C. indicum*, whereas d-limonene and β-copaene were detected exclusively in *C. morifolium* cultivars. Each of the 44 tested genotypes exhibited distinct qualitative and quantitative profiles of terpenoids, both in terms of compounds emitted from and accumulated within the inflorescences. Notably, wild relatives contained significantly higher total concentrations of terpenoids compared to the cultivated *C. morifolium* cultivars [8].

In another study focused on chrysanthemum teas, terpenoids represented the largest share of the total VOCs, particularly in the herbal cultivar ‘Huangju’ (*C. morifolium*) [16]. Similarly, an analysis of inflorescences from 15 Korean Chrysanthemum species—excluding *C. morifolium*—identified 45 volatile compounds, most of which were terpenoids, with camphor present in the highest concentration [7]. In traditional Korean dried *C. morifolium* flower heads used for beverages called gukhwa, chrysantenyl acetate and 2-pinene-4-ol were reported at the highest concentrations, accounting for 43.74% and 27.85% of the total VOCs, respectively [6]. The terpenoid compounds being the major components of the floral scent found in chrysanthemum flowers included camphor, α-pinene, chrysanthenone, myrcene, eucalyptol, verbenone, β-phellandrene, and camphene, with monoterpenes and oxygenated monoterpenoids [22].

In our study, the relative content and diversity of volatile terpenoid compounds varied significantly among the tested *Chrysanthemum* genotypes. The highest total proportion of terpenoids in the volatile fraction was found in CHR 18 (56.8%) and DC26 (56.9%), followed by CD 46 (47.5%) and DC 19 (45.1%). CD 7 exhibited moderate terpenoid content (15.4%), while CH B had a markedly low proportion (0.6%). CHR 18 showed the richest qualitative profile, with 34 distinct terpenoid compounds detected, including the highest level of alcanfor (camphor) (35.7%) and high levels of α-pinene (6.0%), camphene (2.9%), and eucalyptol (3.8%). DC 26 also exhibited a broad terpenoid spectrum (30 compounds), notably rich in chrysanthenone (13.3%), alcanfor (camphor) (11.7%), and eucalyptol (8.2%), as well as triterpenes such as β-amyrin, α-amyrin. CD 46 was similarly diverse (26 compounds) and contained the highest amount of triterpens such as α-amyrin and β-amyrin (6.0% and 3.9%, respectively) and a unique presence of lupeol derivatives (6.9% in total). Noteworthy, CD 46 was also rich in unique non-terpenoid compound 2-methyloctacosan (16.3%). The nonuniformity of produced terpenoids among cultivars in aromatic plants comes from genetic differences, and the unique aroma of chrysanthemum teas originated from different genotypes can be treated as specific “volatile fingerprint” [16].

Three terpenoids: chrysanthenone, cis-verbenol, and verbenone were found exclusively in the hybrid cultivars. Chrysanthenone was particularly abundant in DC 19 and DC 26, reaching 20.5% and 13.3%, respectively. The non-terpenoid compound 3,5-heptadienal-2-ethylidene-6-methyl, also found only in the hybrid group, is likely a product of terpene degradation. Triterpenes were detected exclusively in the hybrid genotypes, specifically in DC 26 and CD 46. CH B was an outlierwith the lowest terpenoid content and diversity (only 5 compounds), indicating its different phytochemical profile, possibly due to processing or cultivar origin.

Overall, CHR 18 and DC 26 were the most terpenoid-rich and chemically diverse genotypes, suggesting a higher potential for bioactivity and aroma. In contrast, CH B exhibited the least complex and least abundant volatile profile. This may be attributed to the fact that CH B was a commercially purchased raw *chrysanthemi flos* product, with an unknown transportation and storage history. Since terpenoids are highly volatile, they are particularly susceptible to evaporation and degradation over time [23]. This highlights the importance of promoting the use of locally grown chrysanthemums for herbal applications, as shorter supply chain can help preserve their chemical integrity and therapeutic potential. Numerous studies have demonstrated the rich terpenoid composition in the inflorescences of species belonging to the *Chrysanthemum* genus. However, comparisons of results—both qualitative and quantitative—are often challenging due to the wide range of factors influencing terpenoid content [5]. These factors include cultivation practices, as well as post-harvest processing and storage conditions of the herbal material [23].

### 2.2. Polyphenolic Composition and Antioxidant Potential of Chrysanthemum Genotypes

Polyphenols are ubiquitous secondary metabolites synthesized via the shikimate and acetate-malonate pathways and contribute to multiple plant physiological processes and stress responses [24]. They function as chemical defenses against herbivores and pathogens, absorb or screen ultraviolet (UV) light, and buffer oxidative damage produced during abiotic stress. For humans, plant polyphenols are invaluable as antioxidants involved in numerous health-protection biochemical processes [25]. They exhibit significant preventive effects against various chronic diseases, including cardiovascular disorders, diabetes, neurodegenerative diseases, and certain types of cancer. Their mechanism of action is primarily associated with the neutralization of reactive oxygen and nitrogen species, modulation of inflammatory pathways, and regulation of gene expression responsible for the oxidative stress response. Research findings indicate that these compounds function not only as direct antioxidants but also as signaling molecules that modulate cellular defense mechanisms and metabolic homeostasis, making them a key component of a health-promoting and preventive diet [26,27,28]. Nonetheless, it should also be taken into account that high intakes of flavonoids may exert deleterious effects due to their diverse pharmacological properties, which may alter drug and amino acid metabolism, modulate the activity of environmental genotoxicants, and affect the activity of other key metabolizing enzymes [29]. However, it should be emphasized that the biological effects of flavonoids are dose-dependent, and adverse effects have been reported primarily at high intake levels. Therefore, this issue represents a general consideration in the application of natural products rather than a contradiction to their beneficial properties.

In the recent study aimed at UV-radiation protective properties of chrysanthemum petals of five local Chinese cultivars it was found that among 22 metabolites identified, there were compounds that significantly correlated with multiple antioxidant and whitening (i.e., UV-protective) indicators. It was shown that not the high antioxidant capacity of extracts, but high flavonoids and phenolics content is positively correlated with the UV protective activity [30]. In another study, both flavonoids and caffeoylquinic acids were highly responsible for the anti-inflammatory effect of *C. indicum* inflorescence through synergetic actions. Moreover, it was stated that 3,5-dicaffeoylquinic acid, luteolin, and linarin were the most important active constituents and could be selected as chemical markers for quality control of *C. indicum flos* [31]. In another study, 63 samples of dried crude *chrysanthemi flos* originating from the USA, China, and Europe, chlorogenic acid, luteolin-7-O-glucoside, apigenin-7-O-glucoside, and 3,5-dicaffeoylquinic acid were detected [4].

The comparative analysis of flavonoids and phenolic acids contents across the six genotypes studied in our experiment also revealed significant variation in their polyphenolic profiles (Table 2 and Figure S1). The detailed MS data are summarized in Table S1. Total flavonoid concentrations ranged from 30.8 mg/g in genotype CD 7 to a maximum of 79.6 mg/g in CHR 18, indicating notable genotype-dependent accumulation. Apigenin 7-O-glucoside emerged as the predominant flavonoid, particularly abundant in CHR 18 (36.9 mg/g) and CD 46 (10.45 mg/g), while luteolin 7-O-glucoside also exhibited substantial levels, especially in DC 19 (19.7 mg/g), DC 26 (17.3 mg/g), and CD 46 (25.6 mg/g). Generally, the hybrid chrysanthemum group was richer in luteolin derivatives, while high apigenin derivatives content was found in tea chrysanthemums (CHR 18 and CH B). Other flavonoids, such as acacetin-7-galactoside and apigenin derivatives, displayed moderate but variable concentrations across genotypes. Notably, all the samples contained linarin (buddleoside) (Table 2), which is claimed to be the characteristic compound to detect *C. indicum* [4,31].

Phenolic acids content similarly varied, with total concentrations ranging from approx. 26 mg/g in CD 7 to 41 mg/g in CHR 18. The phenolic acid’s profile was dominated by dicaffeoyl quinic acid derivatives, particularly 3,5-dicaffeoyl quinic acid, which showed the highest values in CHR 18 (19.6 mg/g) and DC 19 (17.7 mg/g). Chlorogenic acid was another major constituent, present in all genotypes at concentrations between 4.6 and 8.5 mg/g. Overall, the CHR 18 genotype consistently displayed the highest levels of both flavonoids and phenolic acids, suggesting it is a rich source of these bioactive compounds. Conversely, genotype CD 7 showed the lowest total polyphenolics content. The antioxidant activity of dried chrysanthemum inflorescences is a result of their biochemical composition—primarily the content of polyphenol derivatives (including phenolic acids and anthocyanins), as well as carotenoids—and also depends on the drying and storage conditions [32,33].

To comprehensively evaluate the antioxidant potential of the tested chrysanthemum genotypes, several complementary assays were employed. The FRAP and CUPRAC tests were used to assess the reducing power of the extracts, while the DPPH and ABTS assays measured their free radical scavenging capacity. In addition, the iron chelation assay was applied to determine the metal ion-binding ability, which contributes to the prevention of oxidative stress through inhibition of pro-oxidant metal-catalyzed reactions.

The antioxidant potential varied significantly among the tested samples. Hybrids DC 19, CD 46, and DC 26 exhibited the highest reducing capacities in the FRAP and CUPRAC assays, as well as the highest radical-scavenging activity in the DPPH test. Iron-chelating ability was highest in cultivar CHR 18, followed by CH B among the hybrids. Overall, the hybrid genotypes, particularly CD 46 and DC 26, demonstrated superior antioxidant performance, confirming their potential as promising sources of natural antioxidants.

**Figure 1:**
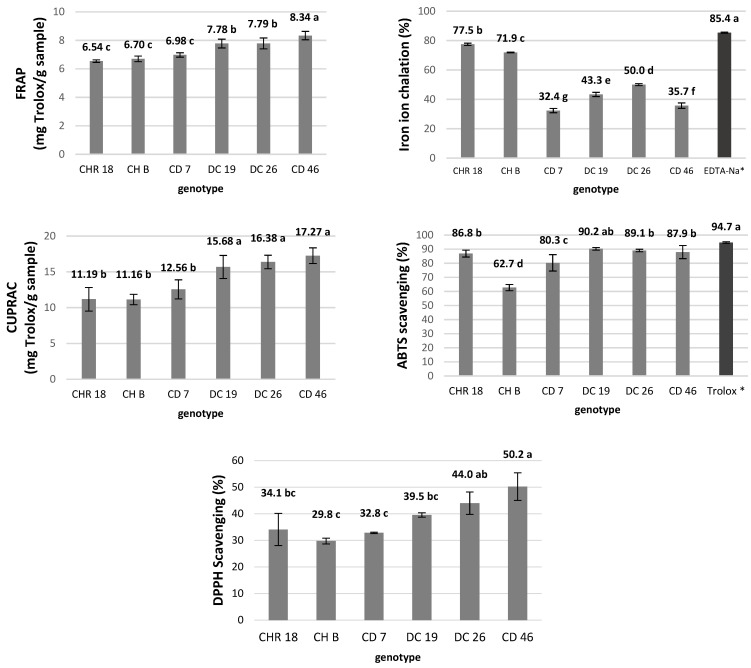
Indication of the antioxidant properties by means of different assays for extracts derived from dry crude inflorescences of the tested genotypes of chrysanthemum. Means ± SD labeled with the same letter do not differ significantly according to Tukey’s HSD test at *p* ≤ 0.05. * EDTA-Na and Trolox were used as reference solutions for ferrosine inhibition (iron ion chelation assay) and ABTS scavenging, respectively.

Interestingly, the highest antioxidant capacity was observed in CD 46, rather than in CHR 18, which had the highest polyphenolic content (Figure 1). This may be attributed to the presence of anthocyanins in the crude material of CD 46, which play an important role in free radical scavenging [34].

**Figure 2:**
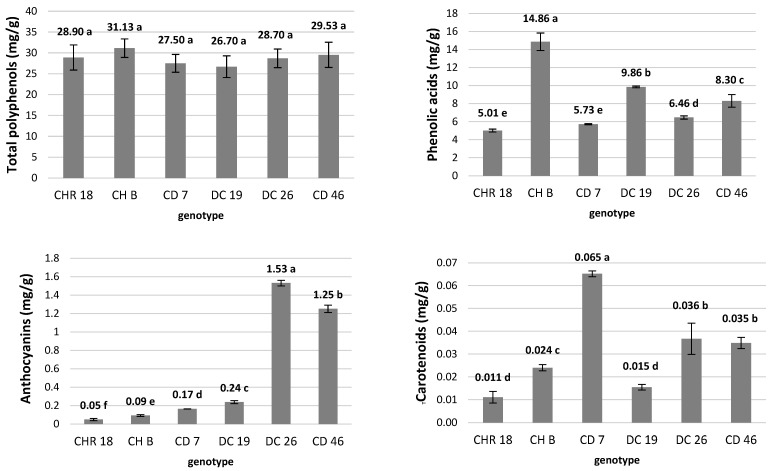
Total polyphenols (expressed as mg of pyrogallol), phenolic acids (expressed as mg of caffeic acid), anthocyanins (expressed as mg of cyanidine-3-glucoside) and carotenoids (expressed as mg of β-carotene) content in dry crude chrysanthemum inflorescences of the tested genotypes. Means ± SD labeled with the same letter do not differ significantly according to Tukey’s HSD test at *p* ≤ 0.05.

As revealed by classical analytical methods (Figure 2) the total polyphenol content (expressed as mg of pyrogallol) did not differ significantly between the tested genotypes and ranged from approx. 27 to 31 mg/g. In contrast, significant differences were observed in phenolic acids content (expressed as mg of caffeic acid). The highest amount was recorded in CH B—a commercial sample of crude inflorescences sold as tea chrysanthemum—with 14.9 mg/g. The lowest content was observed in the Chinese reference cultivar CHR 18 (5.0 mg/g). Among hybrid cultivars, total phenolic acid content ranged from 5.7 mg/g in CD 7 to approx. 10 mg/g in DC 19. These results do not fully correspond to LC-MS analyses, by which only selected polyphenols were indicated. Furthermore, the discrepancy may result from the fact that spectrophotometric assays such as Folin–Ciocalteu measure the overall reducing capacity of the extract, reacting not only with phenolic compounds but also with other reducing substances such as sugars, ascorbic acid, and amino acids [35].

### 2.3. Pigments Composition in Dried Crude Chrysanthemum Inflorescences

The presence of pigments other than chlorophyll in the flower heads of chrysanthemums originates from ligulate florets, disc florets, as well as receptacles and sepals. They are mainly responsible for the inflorescence main color and exhibit other functions related to photoprotection and photosynthesis enhancement [36]. In our experiment, the anthocyanins were detected in all the tested inflorescences (Figure 2); however, their content was clearly associated with flower color. Trace amounts were found even in the pure white CHR 18 genotype and in the pale-colored commercial sample CH B (0.05 and 0.09 mg/g, respectively). The highest anthocyanin content was found in the pink-flowered hybrid DC 26 (1.53 mg/g). It is worth mentioning that all traditional Chinese herbal chrysanthemums are white to yellow color [4].

Similarly, carotenoids were present in all analyzed samples, with significant differences between genotypes. The highest content was detected in the light honey-yellow hybrid CD 7. The hybrids DC 26 and CD 46 followed with 0.036 and 0.035 mg/g, respectively.

The lowest carotenoid content was found in the white-flowered reference cultivar CHR18. It should be noted that carotenoids are present not only in the florets but also in the green parts of the inflorescence, including the sepals and receptacle, which is used in beverage preparation. However, due to the low solubility of carotenoids in water, their availability for consumers in the final infusion may be limited. Nonetheless, since the TCM system utilizes different forms of herbs, carotenoids form dried crude inflorescences may ultimately contribute to positive health effects [37].

### 2.4. Hyaluronidase and Butyrylcholinesterase Inhibition of Chrysanthemum Extracts

Hyaluronidase (HYAL) is an enzyme responsible for the hydrolysis of hyaluronic acid. In humans, it is involved in fertilization and skin aging [38]. HYAL facilitates the penetration of bacteria into the host organism by increasing the permeability of cell membranes and blood vessels. It is also a key component of snake and spider venoms, enhancing the diffusion of toxins into host tissues [39]. Furthermore, hyaluronidase activity is considered to promote the spread of cancer cells through the degradation of hyaluronic acid, which normally stabilizes cell–cell adhesion [40].

Therefore, hyaluronidase inhibitors are regarded as immune-supportive agents that hinder microbial invasion into tissues, delay skin aging by reducing the loss of firmness, and may also inhibit tumor metastasis. Numerous studies have demonstrated the potential of plant extracts as HYAL inhibitors. High inhibitory activity has been reported for pomegranate rind extract—68.5 ± 5.3% inhibition and isolated quercetin from pomegranate peel—90.9 ± 2.7% inhibition [41], *Ravenala madagascariensis*—64.3% [42], and *Artocarpus nobilis* bark—68.6% [43].

**Figure 3:**
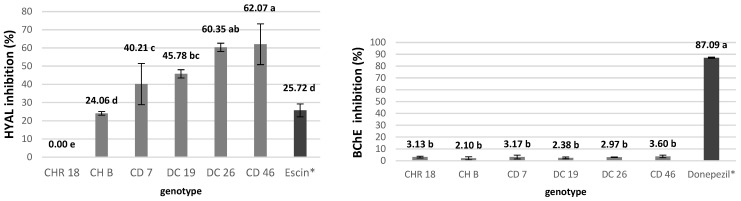
Hyaluronidase (HYAL) and butyrylcholinesterase (BChE) enzyme inhibition capacity of chrysanthemum inflorescence extracts in the tested genotypes. Acetylcholinesterase (AChE) inhibition test detected no activity of chrysanthemum inflorescences extracts in terms of inhibition of AChE. Means ± SD labeled with the same letter do not differ significantly according to Tukey’s HSD test at *p* ≤ 0.05. * Escin and donepezil were used as references for HYAL and BChE, respectively.

To date, chrysanthemum inflorescences have not been investigated for hyaluronidase inhibition. Our results revealed the highest anti-HYAL activity in extracts from hybrid genotype CD 46 (62.1%), while no inhibitory activity was detected in CHR 18 (Figure 3). Extracts from hybrid genotypes showed greater activity than both the commercial *C. morifolium* samples—CH B and CHR 18 (24.1% and 0.0%, respectively) and the reference escin extract (25.7%). The inhibitory effect of plant extracts on hyaluronidase is attributed to flavonoids, which can spontaneously bind to the enzyme—mainly through electrostatic forces and hydrophobic interactions at a single binding site—thereby inactivating it [44]. Since chrysanthemum extracts are not particularly rich in quercetin, as confirmed by LC-MS analyses, the high inhibitory activity observed in hybrids can be attributed to their considerable luteolin derivatives content, with the highest concentration found in CD46—nearly 40 mg/g.

These findings indicate substantial genotypic variation in HYAL inhibition capacity, with *C. morifolium* (CH B, CHR 18) showing lower or absent activity compared to *C. morifolium* × *C. rubellum* hybrids. This important finding highlights the potential of hybrid chrysanthemum inflorescences as novel sources of bioactive compounds with anti-HYAL properties.

The potential neuroprotective activity of *C. morifolium* extracts, as assessed by anti-acetylcholinesterase (AChE) and anti-butyrylcholinesterase (BChE) assays, is not well documented, although several studies have reported AChE and BChE inhibitory effects in *C. indicum*, *C. fontanesii*, and *C. coronarium* [20,21,43,44,45].

In our study, an acetylcholinesterase (AChE) inhibition test detected no activity of the chrysanthemum inflorescences extracts in terms of the inhibition of AChE and only weak BChE inhibition (2.10–3.60%), regardless of genotype (Figure 3). In comparison, donepezil, a common Alzheimer’s disease medication used as a reference, showed 87.09% anti-BChE activity. Thus, crude dried chrysanthemum inflorescences demonstrated no meaningful BChE inhibitory effect.

### 2.5. Correlation Analysis of Phytochemical and Biological Parameters

As indicated by the correlation analysis, anthocyanin content correlated strongly with FRAP, CUPRAC, and DPPH assays (r = 0.73–0.79) (Table 3), suggesting that anthocyanins are major contributors to the overall antioxidant capacity of the tested cultivars. These variations indicate potential differences in health-promoting effects associated with genotype-specific polyphenol accumulation, emphasizing the importance of genetic factors in shaping the phytochemical composition of plant-derived materials [36,45]. Furthermore, the correlation analysis revealed that HYAL inhibition was strongly associated with FRAP, CUPRAC, DPPH, and anthocyanin content (r = 0.65–0.81). Notably, genotypes DC 26 and CD 46 exhibited both the highest anthocyanin levels (1.5 mg/g and 1.3 mg/g, respectively) and the strongest HYAL inhibitory activity (from approx. 60% to 62%, respectively) (Table 3).

## 3. Materials and Methods

### 3.1. Plant Material and Field Cultivation

Hybrid genotypes used in this study were obtained in year 2020 as a result of reciprocal crossing between *Chrysanthemu* × *morifolium* (Ramat.) ‘Donna’ and *Chrysanthemum rubellum* ‘Clara Curtis’. From the total number of 49 F1 hybrids, four were selected concerning the earliness of flowering, namely: CD 7, DC 19, DC 26 and CD 46 and were used in the experiment. Genotype CHR 18 which was used as cultivated reference is declared by the producer to be “traditional Chinese tea chrysanthemum Ju Hua” (https://www.kraeuter-und-duftpflanzen.de, accessed on 30 July 2025) [45]. The phenotype details of cultivated plants, as well as the dates and period of flowering and the temperature distribution details during the cultivation period, are presented in Supplementary Materials (Table S2 and Figure S2).

Vegetative shoot-tip cuttings of hybrid genotypes were harvested from mother plants and rooted in the greenhouse of Faculty of Agriculture and Biotechnology at Bydgoszcz University of Science and Technology, Poland. Cuttings of CHR 18 genotype were harvested from plants which were purchased from the commercial grower (Ruehlemann’s, Uetze, Germany). The rooted vegetative cuttings of tested genotypes, both hybrids and reference, were planted in April 2021 in field conditions in the university garden in Bydgoszcz, Poland, in the native soil enriched with peat-based horticultural substrate (Gramoflor, Bydgoszcz, Poland). To test their winter-hardiness, plants were cultivated from 2021 to 2024 in this location (53.12070° N, 18.00691° E), fertilized twice a year—in the April and August with common garden fertilizer (proportion of N:P:K as 1:0.5:1.4) in the amount of 70 g/m^2^ (Azofoska, Grupa Inco SA, Włocławek, Poland).

### 3.2. Preparation of Crude Plant Material and Basal Methanolic Extract

**Figure 4:**
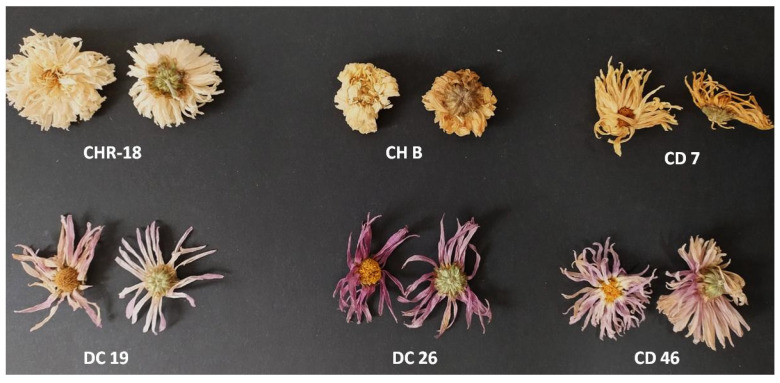
Dry inflorescences of tested chrysanthemum cultivars before grinding. The dry flower heads of CH B were purchased from a commercial company importing the herbal chrysanthemum crude material from China, as indicated on the packaging. The inflorescences of the rest of the tested genotypes were harvested from plants grown in the University’s garden.

The inflorescences (flower heads) were harvested from field grown plants in the September and October 2024, during the fourth year of cultivation. For hybrid chrysanthemums, the flower heads were harvested when the half of the disc florets were abundant in pollen. In the case of genotype CHR 18, the inflorescences were collected when the ray florets half-way from the center were fully open. Flower heads were air-dried in the laboratory oven in 30–35 °C (Figure 4).

For the comparison of field originated plant material with market-available product, dry chrysanthemum flower heads were purchased from the commercial company as “White chrysanthemum—edible flowers” (Winoszarnia, Lodz, Poland) and were incorporated in the analyses (labeled as CH B). The place of origin of these dry inflorescences indicated on the packaging was China.

Crude, dry inflorescences of all tested genotypes were ground in the laboratory mill, sieved at 0.5–1 mm and thus used for the experiments.

Unless otherwise stated, basal extracts were prepared as follows. Using an analytical balance and weighing paper, 300 mg of each tested plant raw material was weighed. The weighed samples were transferred to 50 mL Erlenmeyer flasks, 3 mL of methanol was added, and the flasks were sealed with stoppers and kept in the dark for 24 h. Subsequently, the extracts were filtered through paper filters into previously weighed 50 mL Erlenmeyer flasks. The filtrates were left until complete evaporation of methanol. The flasks were then reweighed, and methanol was added in a proportion of 1 mL of methanol per 25 mg of the remaining residue in order to dissolve the residues.

### 3.3. Phytochemical Characteristics of Tested Inflorescences Extracts

#### 3.3.1. Analyses of Terpenoid Profile with GC-MS

Lyophilized crude extracts obtained from the inflorescences of six tested genotypes were diluted 200-fold with hexane prior to analysis. All samples were filtered through 0.25 μm syringe filters and transferred to chromatographic vials for analysis. Gas chromatography–mass spectrometry (GC–MS) analysis was performed using a Bruker (Billerica, MA, USA) 436-GC gas chromatograph coupled with a Bruker SCION SQ single quadrupole mass spectrometer equipped with an electron ionization (EI) source. Chromatographic and spectrometric parameters were as follows: capillary column: BR-5 (5%-phenyl-95%-dimethylpolysiloxane), 30 m × 0.25 mm i.d., film thickness 0.25 μm; oven temperature program: 60 °C for 4.0 min; ramp at 12 °C/min to 180 °C (hold 0 min), ramp at 8 °C/min to 240 °C (hold 0 min), ramp at 25 °C/min to 300 °C, hold for 4.1 min; injector temperature: 300 °C; split ratio: 1:20; carrier gas: Helium; flow rate: 1.0 mL/min, transfer line temperature: 300 °C, ion source temperature: 220 °C, ionization energy: 70 eV, scan range: *m*/*z* 50–500. Chromatographic peaks were identified using a dual-approach strategy: (1) by comparison of the obtained mass spectra with those from the NIST 11 mass spectral library, and (2) by calculation of Kováts retention indices (RI) in the range of 800–2000, using a standard mixture of n-alkanes (C_8_–C_20_) under identical chromatographic conditions. The contents of identified terpenoids in studied extracts were expressed as relative peak area percentages (%).

#### 3.3.2. Total Anthocyanins, Carotenoids, Polyphenols and Phenolic Acids Content

Total anthocyanins content was determined using modified Harborne method [36,46]. Anthocyanins were extracted from 200 mg of dry inflorescences using 1% HCl in methanol. Absorbance was measured at 530 nm, and total anthocyanin content in the extract [g/L] was calculated and expressed as mg of cyanidin-3-glucoside per 1 g of crude material using the formula:

(1) $$C_{a}\;=\frac{A_{530}}{61.7}$$

Total carotenoids content was determined using modified Wettstein method [36,47]. Carotenoids were extracted from 100 mg of ray florets in 10 mL of acetone. Absorbance was measured at 440 nm. Total carotenoid content [mg/L] was calculated and expressed as mg of β-carotene in 1 g of crude material using the formula:

(2) $$C_{k}\;=\;4.695\;*A_{440}$$

The total polyphenols content was determined using the Folin–Ciocalteu method in accordance with the Polish Pharmacopoeia, 6th ed. [48]. A 0.5 g sample of dried, powdered plant material was extracted with 150 mL of distilled water by boiling for 30 min. After cooling and filtration, the extract was diluted, and a 2 mL aliquot was reacted with Folin–Ciocalteu reagent, distilled water, and sodium carbonate solution (290 g/L) in a 25 mL flask. The mixture was incubated in the dark for 30 min, and absorbance was measured at 760 nm. A pyrogallic acid standard curve was used for quantification. Results were calculated according to equation and finally expressed as mg of pyrogallic acid per 1 g of crude material.

(3) $$X=\frac{62.5*m_{2}*A_{1}}{m_{1}*A_{2}}$$

X—polyphenol content expressed as pyrogallol [%], A_1_—absorbance value of the tested solution, A_2_—absorbance value of the reference pyrogallol solution, m_1_—mass of the weighed plant material [g], m_2_—mass of the weighed pyrogallol [g].

The total phenolic acid content was determined according to the Polish Pharmacopoeia, 6th edition [48], using the Arnow method and expressed as caffeic acid equivalents. Approximately 1 g of powdered plant material (sieved through a 0.315 mm mesh) was extracted twice with distilled water, filtered, and the combined extracts were diluted to 50 mL. A 1 mL aliquot was reacted with HCl, Arnow’s reagent, and NaOH, and absorbance was measured at 490 nm against a reagent blank. Results were calculated according to equation.

(4) $$X=\frac{A*1.7544}{m}$$

X—phenolic acids content expressed as caffeic acid [%], A—absorbance value of the tested solution, m—mass of the weighed plant material [g]. Results were finally expressed as mg of caffeic acid per 1 g of crude material.

#### 3.3.3. Detailed Analyses of Phenolic Acids and Flavonoids Compounds with Liquid Chromatography-High Resolution Mass Spectrometry (LC-HRMS)

Lyophilized, dry crude material from the inflorescences of six tested genotypes was used for the experiment. Each analysis was performed two times. LC-HRMS analysis was performed using ultra-high performance liquid chromatograph (UHPLC) Infnity Series II with a DAD detector and Agilent 6224 ESI/TOF mass detector (Agilent Technologies, Santa Clara, CA, USA) with the column: Kinetex C18 (Phenomenex, Torrance, CA, USA), 150 mm in length, 2.1 mm inner diameter, and a particle size of 1.7 µm. Chromatographic conditions were as follows: The column thermostat: 30 °C. The flow rate: 0.2 mL/min. The mobile phase consisted of water with 0.05% formic acid (solvent A) and acetonitrile with 0.05% formic acid (solvent B). Gradient elution was performed according to the following program: 0–8 min from 98% A to 93% A, 8–15 min from 93% A to 88%A, 15–29 min from 88% A to 85% A, 29–40 min from 85% A to 80% A, 40–80 min from 80% A to 55% A. LC–HRMS conditions: drying gas temperature 325 °C, drying gas flow 8 L min-1, nebulizer pressure 30 psi, capillary voltage 3500 V, and skimmer 65 V. Voltage on the fragmentator was 170, 200 V, and 280 V. Various ionization energies were used to explore the fragmentation patterns and enhance the identification of compounds. Ions were acquired in the range of 100 to 1200 *m*/*z* in negative mode. Identification was based on comparison with standard or based on references [49,50].

### 3.4. Evaluation of Antioxidant Activity of Chrysanthemum Inflorescences Extracts

#### 3.4.1. Ferric Ion Reducing Antioxidant Power (FRAP) Assay

The FRAP assay was carried out by mixing extracts at a concentration of 1.0 mg/mL with 290 μL of a working solution consisting of acetate buffer (15 mL), TPTZ solution (1.5 mL), and FeCl_3_·4H_2_O (1.5 mL). The reaction mixture was allowed to incubate for 30 min, after which the absorbance was measured at 600 nm. Trolox and BHA were used as positive controls. The results of the assay were expressed as milligrams of Trolox per gram of sample (mg Trolox/g sample) [51].

#### 3.4.2. Cupric Ion Reducing Antioxidant Capacity (CUPRAC) Assay

The CUPRAC assay was carried out by combining 10 μL of the extract (at a concentration of 1.0 mg/mL) with 80 μL of acetate buffer (pH 7.0), 50 μL of neocuproine (7.5 mM), and 50 μL of CuCl_2_ (10 mM). The mixture was incubated for 5 min, after which the absorbance was measured at 450 nm. The results of the CUPRAC assay were then expressed as milligrams of Trolox equivalent per gram of sample (mg Trolox/g sample) [51].

#### 3.4.3. ABTS Assay for Free Radical Scavenging Activity

The experiment was performed using a 96-well plate format. 1.0 mg/mL were tested. In brief, 10 μL of the extract was combined with 190 μL of ABTS solution prepared with potassium persulfate. For the background control, 10 μL of pure DMSO (100%) was mixed with 190 μL of ABTS solution. Ascorbic acid, BHA, and Trolox served as reference antioxidants. The plate was incubated in darkness for 30 min before measuring absorbance at 405 nm [51]. The following formula was used to calculate the results obtained:

(5) $${INH}_{ABTS}=(\frac{A_{C}-A_{S}}{A_{C}})*100\%$$

A_S_—absorbance of the sample + ABTS;

A_C_—absorbance of the ABTS.

#### 3.4.4. The DPPH Scavenging Assay

The modified radical scavenging method using the DPPH (2,2-diphenyl-1-picrylhydrazyl) was applied [51,52]. A 0.1 mM solution of DPPH in methanol was prepared and kept in the dark. To determine scavenging activity, 7.7 µL of concentrated methanolic plant extract (25 mg/mL) was mixed with 3.0 mL of DPPH solution. The mixture was incubated in the dark at room temperature for 30 min. After incubation, the absorbance was measured at 515 nm against a methanol blank. A solution without extract was used as the control. The radical scavenging activity (RSA) was calculated using the following formula:

(6) $${INH}_{DPPH}=(\frac{A_{C}-A_{S}}{A_{C}})*100\%$$

A_S_—absorbance of the sample + DPPH;

A_C_—absorbance of the DPPH.

#### 3.4.5. Iron (II) Ion Chelation Assay

The assay was carried out using a 96-well plate. 1.0 mg/mL were tested. Each well contained 140 μL of methanol, 5 μL of FeCl_2_ solution, and 100 μL of the extract. Control samples were prepared using 135 μL of methanol, 5 μL of FeCl_2_ solution, and 100 μL of pure DMSO. After an initial incubation at 25 °C for 5 min., 5 μL of ferrozine solution was added to each well. The plate was further incubated for 10 min at 25 °C before measuring absorbance at 517 nm. The obtained data were processed using a specific formula [53]:

(7) $${INH}_{Chel.}=(\frac{A_{C}-A_{S}}{A_{C}})*100\%$$

A_S_—absorbance of the sample + Ferrozine + Fe^2+^;

A_C_—absorbance of the Ferrozine + Fe^2+^.

### 3.5. Hyaluronidase (HYAL) and Butyrylocholinesterase (BChE) Inhibition Capacity of Chrysanthemum Inflorescences

Hyaluronidase inhibition assays using the bovine enzyme were performed in 96-well microplates according to a modified procedure [54]. The assay relied on the precipitation of unhydrolyzed hyaluronic acid by cetyltrimethylammonium bromide (CTAB) to determine the inhibitory activity of the tested extracts and compounds. The samples were dissolved in 10% aqueous DMSO. In each well, 15 μL of extract solution (10.0, 1.0, or 0.1 mg/mL) was combined with 15 μL of acetate buffer (pH 5.35), 25 μL of incubation buffer (pH 5.35, containing 0.01% BSA and 0.45% NaCl), and 25 μL of enzyme solution (30 U/mL in the same incubation buffer). The mixture was incubated at 37 °C for 10 min, after which 25 μL of hyaluronic acid solution (0.3 mg/mL in acetate buffer, pH 5.35) was added. The plates were subsequently incubated for another 45 min at 37 °C. To precipitate the non-hydrolyzed hyaluronic acid, 200 μL of 2.5% CTAB was added to each well, and the mixture was kept at 25 °C for 10 min. The absorbance was then measured at 600 nm to assess the extent of complex formation. The degree of inhibition was determined by comparing the absorbance of the control sample without inhibitor (A_C_) and the blank sample without enzyme (A_T_). The percentage of hyaluronidase inhibition was calculated using a standard equation, with escin serving as a positive control.

(8) $${HYAL}_{inh}=\frac{A_{S}-A_{C}}{A_{T}-A_{C}}*100\%$$

A_S_—absorbance of the HA + sample + enzyme;

A_C_—absorbance of the HA + enzyme;

A_T_—absorbance of the HA + sample.

The activities of butyrylcholinesterase (BChE) were determined using a modified version of Ellman’s method [54]. Briefly, 5 μL of the extract was mixed with 45 μL of AChE or BChE enzyme solution (0.4 U) and incubated for 15 min. Following incubation, 150 μL of a reaction mixture containing buffer, acetylcholine, and DTNB in a 308:2:1 ratio was added. The absorbance was recorded immediately (time 0) and again after 10 min at 405 nm. Donepezil served as the reference inhibitor. The percentage of enzyme inhibition was calculated according to the following equation:

(9) $${BChE}_{inh}=(1-\frac{A_{S}}{A_{C}})*100\%$$

A_S_—absorbance of the substrate + sample + enzyme;

A_C_—absorbance of the substrate + enzyme.

### 3.6. Experimental Design and Statistical Methods

For the total anthocyanin, carotenoid, polyphenol, and phenolic acid contents, as well as for the antioxidant and enzyme activity assays, the experiments were performed in three replicates and were arranged in a completely randomized design. At least three measurements were taken for each replicate. One-way ANOVA was performed to detect significant effects, and differences between means were compared using Tukey’s HSD test at *p* ≤ 0.05. To verify the significance of correlations between selected groups of compounds’ antioxidant and enzyme activities, Pearson’s correlation coefficients were calculated at *p* < 0.05. Data analyses were performed using the Statistica 13.3 software package (TIBCO Software Inc., Palo Alto, CA, USA).

## 4. Conclusions

Our study demonstrated that chrysanthemum inflorescences represent a valuable plant material rich in bioactive phytochemicals, including phenolic acids, flavonoids, and terpenoids, which may contribute to human health through their antioxidant and enzyme-inhibitory activities. The beneficial phytochemical properties of the inflorescences of *C. morifolium* and *C. rubellum* chrysanthemum hybrids, combined with their potential for cultivation in local European markets, open up a new direction in chrysanthemum production, previously unexplored in Europe. The *C. rubellum* genetic component in the tested hybrids contributed winter hardiness, the unique and distinctive polyphenol and terpenoid profiles, the pigmentation of the crude plant material, and ultimately, antioxidant properties comparable to, or even exceeding, those of pure *C. morifolium* genotypes. Moreover, the hybrid chrysanthemums exhibited markedly higher hyaluronidase inhibition activity—a novel finding in the field of chrysanthemum phytochemistry. Extraordinary phytochemical properties, coupled with an attractive appearance and frost resistance, make *C. morifolium × C. rubellum* hybrids highly promising plant material for both herbal and landscape applications.

## Figures and Tables

**Table 1:** The relative content of terpenoid and some non-terpenoid * compounds (expressed as relative peak area percentages, %) in the total volatile organic compounds (VOC) profile obtained from hexane extract in GC-MS from crude material of dry inflorescences of six tested chrysanthemum genotypes. RT—retention time.

No.	RT [min]	Component	CHR 18	CH B	CD 7	DC 19	DC 26	CD 46
1	5.63	Tricyclene	0.104			0.035		
2	5.69	α-Thujene	0.111				0.097	
3	5.85	α-Pinene	6.067	0.062	0.386	0.529	4.032	0.309
4	6.20	Camphene	2.927	0.038	0.350	0.362	1.220	0.451
5	6.64	Sabinene	0.435		0.041		0.194	
6	6.75	β-Pinene	0.221		0.035	0.037	0.173	0.028
7	6.95	β-Myrcene	0.039					
8	7.04	Pseudocumene *				0.174	0.055	
9	7.61	o-Cymene *	0.064		0.033	0.755	0.544	0.262
10	7.69	Limonene	0.304					
11	7.72	β-Phellandrene	0.974					
12	7.76	Eucalyptol	3.757		1.596	10.040	8.193	3.430
13	8.17	γ-Terpinene		0.109				
14	8.40	β-Terpineol	0.474		0.350	0.298	0.907	0.240
15	8.66	Linalool oxide	0.115				0.243	
16	8.84	Linalool	0.282		0.269	0.102		
17	8.90	cis-Sabinene hydrate	0.405		0.245			0.262
18	8.94	2H-Pyran-3(4H)-one.6-ethenyldihydro- 2.2.6-rimethyl *	0.354				0.254	
19	9.00	trans-3-Caren-2-ol *	0.224		0.303			0.112
20	9.22	Chrysanthenone			4.246	20.470	13.260	3.489
21	9.30	Camphenol	0.085				0.059	
22	9.53	Isopinocarveol	0.063					
23	9.62	(-)Alcanfor	35.720	0.342	6.046	3.932	11.740	4.127
24	9.83	cis-Verbenol			0.234	0.310	3.049	0.176
25	9.94	(+)Borneol				0.135		
26	9.98	endo-Borneol	0.875		0.169	0.049	0.343	0.032
27	10.09	Terpinen-4-ol	0.045			0.327	0.193	0.085
28	10.20	(-)Carvone				0.047		
29	10.28	α-Terpineol	0.467		0.222	0.564	0.738	0.201
30	10.48	Verbenone			0.023	0.186	0.144	0.041
31	10.61	trans-Carveol	0.153			0.058	0.069	
32	10.74	trans-Chrysanthenyl acetate				0.732	2.679	
33	10.83	D-Verbenone				0.142	0.231	
34	11.09	cis-chrysanthenyl acetate			0.030			
35	11.24	2.5-Bomanedione *						0.133
36	11.46	Bornyl acetate	0.799	0.066		0.052	0.224	
37	11.49	(-)-Myrtenol				0.096		0.024
38	11.87	1-Hexen-3-yne 2.5.5-trimethyl *						0.417
39	11.88	1.3-Cyclopentadiene 5-(1.1-dimethylethyl) *				2.875		
40	11.95	3.5-Heptadien-2-ol. 2.6-dimethyl *						0.155
41	11.99	3.6-Nonadien-1-ol. *			0.119			
42	12.00	trans-Carvyl propionate	0.036					
43	12.17	Eucarvone					0.064	
44	12.73	3.5-Heptadienal. 2-ethylidene-6-methyl *			0.321	2.295	5.025	0.400
45	13.12	Caryophyllene	0.134		0.032			
46	13.36	β-Farnesene	0.083					
47	13.71	α-Curcumene				0.256		
48	13.99	β-Bisabolene	0.123		0.018			
49	14.05	α-Bergamotene	0.046					
50	14.42	dihydro-β-Agariofuran				0.062	0.080	
51	14.52	Neroidol					0.056	
52	14.84	(-)Spathulenol				0.024		
53	14.91	Caryophyllene oxide	0.146			0.101		0.102
54	15.73	α-Acorenol	0.161		0.354	0.093	0.328	
55	15.94	α-Bisabolol	0.105					
56	18.00	trans-2-alpha-Bisabolene epoxide	0.895				0.145	
57	24.39	β-Amyrin					0.379	3.889
58	25.09	α-Amyrin					2.175	6.006
59	25.63	2-methyloctacosane *						16.310
60	26.52	Lupeol						2.960
61	26.72	Lupeol acetate						3.893
	sum of terpenoid compounds in total VOC (%)		56.8	0.6	15.4	45.1	56.9	47.5
total number of different terpenoid compounds			34	5	22	30	30	26

* Volatile compounds that do not belong to terpenoids but are often present in plants.

**Table 2:** The flavonoids and phenolic acids content (mg/g) in dry extracts derived from dry inflorescences of tested chrysanthemum genotypes, detected and measured with LC-HRMS. Data presented are means of three repetitions ± SD.

	Genotype:	CHR 18	CHB	CD7	DC19	DC26	CD 46
Flavonoids (mg/g)							
1.	Acacetin	2.34 ± 0.09	0.16 ± 0.01	0.19 ± 0.01	0.30 ± 0.01	0.37 ± 0.02	0.19 ± 0.01
2.	Acacetin 7-glucuronide	0.80 ± 0.01	0.44 ± 0.02	0.22 ± 0.02	not detected	0.44 ± 0.01	0.28 ± 0.02
3.	Acacetin-7-galactoside	8.08 ± 0.35	0.42 ± 0.02	0.41 ± 0.02	0.67 ± 0.03	1.06 ± 0.06	0.37 ± 0.01
4.	Apigenin	0.40 ± 0.01	0.16 ± 0.01	0.18 ± 0.01	2.20 ± 0.08	0.28 ± 0.01	1.53 ± 0.06
5.	Apigenin 7-O-rutinoside	1.46 ± 0.07	1.40 ± 0.05	0.24 ± 0.01	0.72 ± 0.01	0.83 ± 0.02	0.56 ± 0.01
6.	Apigenin 7-O-glucoside	36.9 ± 0.08	13.5 ± 0.21	1.68 ± 0.04	6.26 ± 0.24	6.62 ± 0.04	10.5 ± 0.28
7.	Apigenin 7-O-glucuronide	0.94 ± 0.04	1.55 ± 0.05	1.70 ± 0.01	0.60 ± 0.01	1.33 ± 0.01	0.86 ± 0.03
8.	Apigenin 7-O-malonylglucoside	2.25 ± 0.11	0.58 ± 0.01	0.20 ± 0.01	0.38 ± 0.01	0.42 ± 0.02	0.83 ± 0.02
9.	Apigenin 7-O-malonylglucoside	16.3 ± 0.53	2.96 ± 0.09	0.82 ± 0.05	1.97 ± 0.08	2.36 ± 0.08	5.02 ± 0.08
10.	Luteolin	0.37 ± 0.02	0.75 ± 0.03	1.19 ± 0.06	8.49 ± 0.47	1.00 ± 0.01	4.09 ± 0.16
11.	Luteolin 7-O-rutinoside	0.55 ± 0.01	1.60 ± 0.01	0.91 ± 0.03	1.54 ± 0.06	2.36 ± 0.07	1.18 ± 0.10
12.	Luteolin 7-O-glucoside	3.90 ± 0.21	11.0 ± 0.36	9.71 ± 0.38	19.7 ± 0.81	17.3 ± 0.54	25.6 ± 0.73
13.	Luteolin 7-O-glucuronide	0.79 ± 0.03	6.16 ± 0.25	10.0 ± 0.39	2.44 ± 0.53	6.22 ± 0.32	5.94 ± 0.12
14.	Luteolin 7-O-malonylglucoside	0.72 ± 0.01	0.72 ± 0.01	1.02 ± 0.10	1.32 ± 0.07	1.60 ± 0.02	3.17 ± 0.02
15.	Buddleoside (linarin)	2.74 ± 0.09	1.13 ± 0.06	1.23 ± 0.05	2.74 ± 0.09	1.63 ± 0.07	0.83 ± 0.04
16.	Diosmin	0.35 ± 0.02	1.16 ± 0.07	0.98 ± 0.05	1.69 ± 0.06	0.80 ± 0.01	0.62 ± 0.03
17.	Diosmetin	0.05 ± 0.01	0.05 ± 0.01	0.04 ± 0.01	0.53 ± 0.05	0.04 ± 0.01	0.06 ± 0.01
18.	Quercetin 7-glucoside	0.70 ± 0.02	0.01 ± 0.01	0.06 ± 0.01	0.12 ± 0.01	0.07 ± 0.01	0.05 ± 0.01
Total content of flavonoids (mg/g):		79.6	43.8	30.8	51.7	44.8	61.6
Phenolic acids (mg/g)							
1.	Protocatechuic acid	0.06 ± 0.01	0.06 ± 0.01	0.06 ± 0.01	0.26 ± 0.02	0.07 ± 0.00	0.08 ± 0.01
2.	Neochlorogenic acid	0.29 ± 0.02	0.37 ± 0.01	0.16 ± 0.01	0.40 ± 0.01	0.19 ± 0.01	0.16 ± 0.01
3.	Chlorogenic acid	7.87 ± 0.14	7.48 ± 0.14	6.79 ± 0.01	4.59 ± 0.15	8.49 ± 0.22	7.30 ± 0.28
4.	Caffeic acid	0.15 ± 0.01	0.09 ± 0.01	0.14 ± 0.01	1.04 ± 0.07	0.09 ± 0.01	0.18 ± 0.01
5.	Dicaffeoyl quinic acid	7.00 ± 0.08	4.39 ± 0.18	4.68 ± 0.06	7.64 ± 0.28	4.40 ± 0.15	6.31 ± 0.22
6.	3,4-Dicaffeoyl quinic acid	1.68 ± 0.05	1.33 ± 0.06	0.97 ± 0.03	1.23 ± 0.06	1.22 ± 0.01	0.91 ± 0.01
7.	3,5-Dicaffeoyl quinic acid	19.6 ± 0.18	8.81 ± 0.12	11.6 ± 0.29	17.7 ± 0.10	11.2 ± 0.04	9.99 ± 0.02
8.	4,5-Dicaffeoyl quinic acid	4.50 ± 0.18	8.03 ± 0.04	1.61 ± 0.03	2.84 ± 0.16	1.86 ± 0.19	1.12 ± 0.10
Total content of phenolic acids (mg/g)		41.1	30.6	26.0	35.6	27.5	26.1

**Table 3:** Correlation matrix between antioxidant properties, secondary metabolites content and enzyme inhibition activity in six tested chrysanthemum inflorescences extracts. Pearson’s coefficients labeled with * are significant at *p* < 0.05.

	FRAP	CUPRAC	Fe Chelation	ABTS	DPPH	Antho- Cyanins	Carote- Noids	Poly- Phenols	Phenolic Acids	HYAL	BChE
FRAP	1.000										
CUPRAC	0.827 *	1.000									
Fe chelation	−0.656 *	−0.609 *	1.000								
ABTS	0.498 *	0.562 *	−0.396 *	1.000							
DPPH	0.877 *	0.782 *	−0.485 *	0.564 *	1.000						
anthocyanins	0.732 *	0.744 *	−0.401 *	0.429 *	0.787 *	1.000					
carotenoids	0.101	0.092	−0.674 *	−0.089	0.016	0.204	1.000				
polyphenols	−0.067	−0.112	0.295	−0.312	0.033	0.051	−0.131	1.000			
phenolic acids	−0.068	−0.140	0.259	−0.703 *	−0.234	−0.211	−0.276	0.323	1.000		
HYAL	0.806 *	0.800 *	−0.755 *	0.340 *	0.653 *	0.777 *	0.386 *	−0.099	0.014	1.000	
BChE	0.036	0.043	−0.207	0.133	0.048	0.141	0.303 *	−0.336 *	−0.281	0.185	1.000

## Data Availability

Raw, unprocessed data are available from corresponding author (N.M.) on reasonable request.

## Supplementary Materials

The following supporting information can be downloaded at https://www.mdpi.com/article/10.3390/molecules31010172/s1: Table S1: Qualitative composition of flavonoids and phenolic acids identified in the six studied *Chrysanthemum* genotypes. Figure S1: Representative LC–MS chromatograms of extracts from the six studied *Chrysanthemum* genotypes. Table S2: Growth characteristics of the tested chrysanthemums cultivated in the field conditions. Figure S2: Temperature distribution (daily average, daily minimum, and the lowest temperature in the month) during the cultivation period (from April 2021 to November 2024) of the studied chrysanthemums at their growing site in the university garden of Bydgoszcz University of Science and Technology, Poland (location: 53.12070° N, 18.00691° E).
