# Supplementary material for: Unlocking the Functional Value of European-Originated Chrysanthemum Hybrids: Phytochemical and Bioactivity Assessment
Source: Molecules. 2026 Jan 1;31(1):172. doi: 10.3390/molecules31010172 (PMC12787676; doi:10.3390/molecules31010172)
Supplement: Supplementary file 1 [file molecules-31-00172-s001.zip › molecules-4030236-supplementary-revised(1).pdf]

Supplementary material

Unlocking the Functional Value of European-Originated *Chrysanthemum* Hybrids: Phytochemical and Bioactivity Assessment

Natalia Miler[1]*, Maciej Balcerek[2], Jakub Gębalski[2], Anita Woźny[1], Magdalena Wójciak[3], Ireneusz Sowa[3] and Daniel Załuski[2]

Table S1. Qualitative composition of flavonoids and phenolic acids identified in the six studied *Chrysanthemum* genotypes.

Rt (min)	[m/z-H]-	ppm	Formula	Compound
6.72	299.07802 (137)	2.60	$C_{13}H_{16}O_8$	Hydroxybenzoic acid glucoside
7.90	315.07321	3.34	$C_{13}H_{16}O_9$	Dihydroxybenzoic acid hexoside
8.71	153.01955	1.41	$C_7H_6O_4$	**Protocatechuic acid***
10.18	203.08333	3.57	$C_{11}H_{12}N_2O_2$	Tryptophan*
11.21	353.08819 (179,191)	1.09	$C_{16}H_{18}O_9$	**Neochlorogenic acid***
12.78	341.08778 (179)	-0.08	$C_{15}H_{18}O_9$	Caffeoylglucose
15.21	341.08795 (179)	0.42	$C_{15}H_{18}O_9$	Caffeoylglucose
16.20	353.08856 (191, 179)	2.13	$C_{16}H_{18}O_9$	**Chlorogenic acid***
16.96	179.03568	3.88	$C_9H_8O_4$	**Caffeic acid***
18.84	387.16698	2.38	$C_{18}H_{28}O_9$	unknown
28.99	449.10928 (287)	0.77	$C_{21}H_{22}O_{11}$	Unknown flavonoid
31.79	609.14638 (300)	0.45	$C_{27}H_{30}O_{16}$	**Quercetin 7-O-rutinoside***
32.41	593.15058 (285)	-1.03	$C_{27}H_{30}O_{15}$	**Luteolin 7-O-rutinoside**
33.07	447.09481 (285)	3.40	$C_{21}H_{20}O_{11}$	**Luteolin 7-O-glucoside***
33.49	461.07198	-1.23	$C_{21}H_{18}O_{12}$	**Luteolin 7-O-glucuronide**
37.07	515.12003 (353)	1.03	$C_{25}H_{24}O_{12}$	**3,4-Dicaffeoyl quinic acid***
38.60	577.15685 (269)	0.99	$C_{27}H_{30}O_{14}$	**Apigenin 7-O-rutinoside**
38.80	515.12069 (353)	2.31	$C_{25}H_{24}O_{12}$	**3,5-Dicaffeoyl quinic acid***
39.39	515.11967 (353)	0.33	$C_{25}H_{24}O_{12}$	**Dicaffeoyl quinic acid**
39.82	431.09905	1.57	$C_{21}H_{20}O_{10}$	**Apigenin 7-O-glucoside***
40.68	445.0781 (269)	1.04	$C_{21}H_{18}O_{11}$	**Apigenin 7-O-glucuronide***
41.38	607.16568	-1.91	$C_{28}H_{32}O_{15}$	**Diosmin***
42.64	515.12057 (353)	2.07	$C_{25}H_{24}O_{12}$	**4,5-Dicaffeoyl quinic acid***
42.82	533.09359 (489,285)	-0.17	$C_{24}H_{22}O_{14}$	**Luteolin 7-O-malonylglucoside**
42.99	461.10911 (299)	0.38	$C_{22}H_{22}O_{11}$	Diosmetin glucoside
46.60	517.09961 (473,269)	1.63	$C_{24}H_{22}O_{13}$	**Apigenin 7-O-malonylhexoside**
48.30	517.09832 (473,269)	-0.86	$C_{24}H_{22}O_{13}$	**Apigenin 7-O-malonylhexoside**
48.65	575.14273 (269,473)	3.65	$C_{27}H_{28}O_{14}$	Apigenin derivative (only 1,5)
49.07	637.17715 (283,269)	-0.41	$C_{36}H_{48}O_{10}$	Acacetin derivative (only 3,4,5,6)
49.95	503.1179 (299)	-3.17	$C_{24}H_{23}O_{12}$	Diosmetin derivative
51.35	637.17687 $[M+HCOO]^-$ (591, 283)	-0.91	$C_{28}H_{32}O_{14}$	**Buddleoside (linarin)**
51.76	285.04163	4.08	$C_{15}H_{10}O_6$	**Luteolin***
53.98	491.11971 $[M+HCOO]^-$ (445, 283,268)	0.43	$C_{22}H_{22}O_{10}$	**Acacetin-7-galactoside**
54.51	473.1092 (283,269)	0.56	$C_{23}H_{22}O_{11}$	Acacetin derivative
54.81	459.09379 (283,269)	1.10	$C_{22}H_{20}O_{11}$	**Acacetin 7-glucuronide**
58.61	269.04524	-1.14	$C_{15}H_{10}O_5$	**Apigenin***

58.98	283.06248 (268)	4.52	$C_{16}H_{12}O_5$	**Acacetin***
60.11	299.05653	1.39	$C_{16}H_{12}O_6$	**Diosmetin***

* Confirmed by the standard; when the standard was unavailable, quantification was based on the calibration curve for the appropriate aglycone; compounds in bold were quantified.

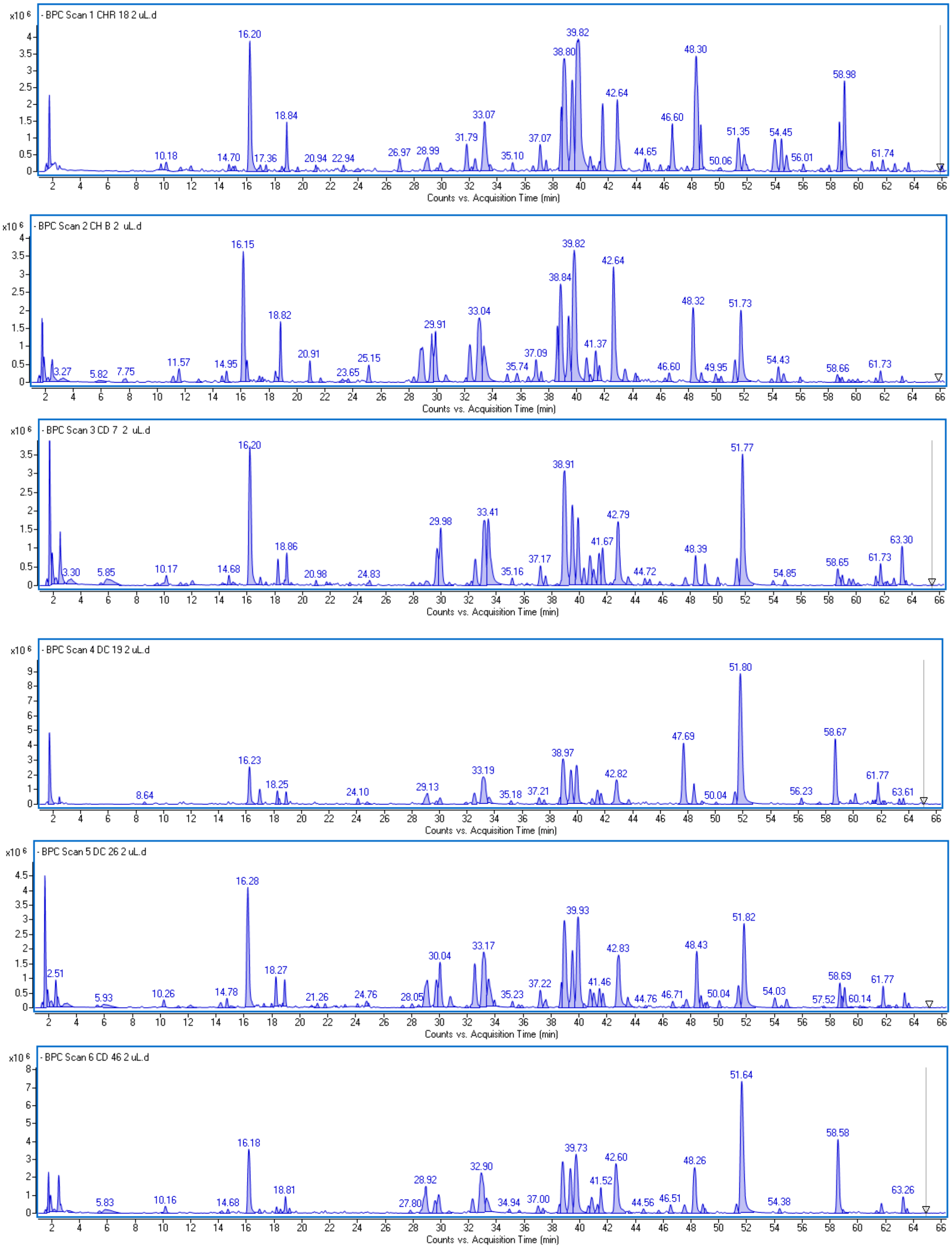

Figure S1. Representative LC–MS chromatograms of extracts from the six studied *Chrysanthemum* genotypes.

Table S2. Growth characteristics of the tested chrysanthemums cultivated in the field conditions.

genotype denomination/ dominant color of inflorescence	photo	origin	earliness; flowering period	height/width of single plant	habit, evaluated from the second year of cultivation
CHR 18 white	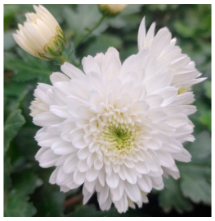	commercial company, declared as traditional Chinese Ju Hua tea chrysanthemum	late; 10th October – 10th November	60-80 cm/ 25-30 cm	not bushy – forms several long single stems
CD 7 yellow	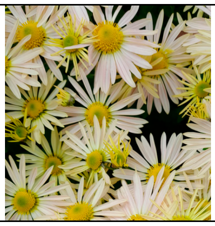	hybrid, progeny of *C. rubellum* ' Clara Curtis'× *C.morifolium* 'Donna'	very early; 1st September – 15th October	30-40 cm/ 30-40 cm	bushy, forms 15-20 branching suckers
DC 19 pink	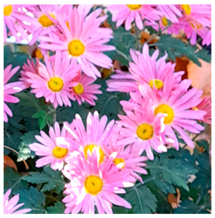	hybrid, progeny of *C. morifolium* 'Donna' × *C. rubellum* ' Clara Curtis'	very early; 1st September – 15th October	50-60 cm/ 50-60 cm	very bushy, forms abundance (more than 20) of branching suckers
DC 26 dark pink	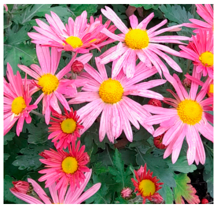	hybrid, progeny of *C.morifolium* 'Donna' × *C. rubellum* ' Clara Curtis'	Early; 15th September – 30th October	40-50 cm/ 50-60 cm	very bushy, forms abundance (more than 20) of branching suckers
CD 46 salmon-pink	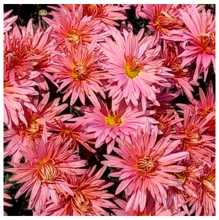	hybrid, progeny of *C. rubellum* ' Clara Curtis'× *C. morifolium* 'Donna'	Early; 15th September – 30th October	60-70 cm/ 50-60 cm	very bushy, forms abundance (more than 20) of branching suckers

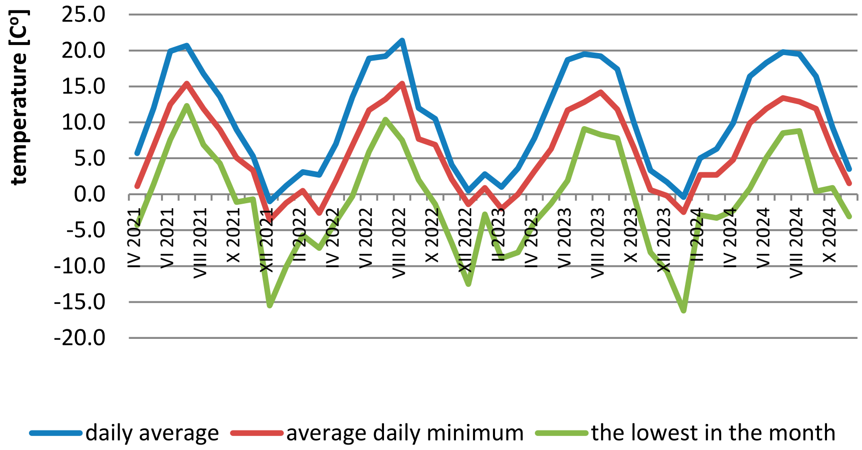

Figure S2. Temperature distribution (daily average, daily minimum, and the lowest temperature in the month) during the cultivation period (from April 2021 to November 2024) of the studied chrysanthemums at their growing site in the university garden of Bydgoszcz University of Science and Technology, Poland (location: 53.12070° N, 18.00691° E)
